# Searching for atrial fibrillation post stroke: is it time for digital devices?

**DOI:** 10.3389/fcvm.2023.1212128

**Published:** 2023-07-27

**Authors:** Olivier Piot, Céline Guidoux

**Affiliations:** ^1^ Department of Cardiac Arrhythmia, Centre Cardiologique du Nord, Saint-Denis, France; ^2^ Department of Neurology and Stroke Unit, Bichat Hospital, Assistance Publique–Hôpitaux de Paris, Paris, France

**Keywords:** atrial fibrillation, stroke, screening, digital devices, connected tools

## Abstract

The detection of atrial fibrillation (AF) in patients with cryptogenic stroke (CS) is an essential part of management to limit the risk of recurrence. However, in practice, not all patients who need AF screening are screened, or are screened with significant delays. The disparities of access to examinations, their costs as well as the increasing workload require an evolution of practices both in terms of organization and the type of equipment used. The ubiquity and ease of use of digital devices, together with their evaluation in large population and their expected lower cost, make them attractive as potential alternatives to current equipment at all stages of patient management. However, reliability and accuracy of each digital device for the detection of paroxysmal AF in CS patients should be established before consideration for inclusion in clinical practice. The aim of this short analysis is therefore to review the current practical issues for AF detection in post stroke patients, the potential benefits and issues using digital devices in stroke patients and to position the different digital devices as alternative to standard equipment at each stage of stroke patient pathway. This may help to design future studies for the evaluation of these devices in this context. Under this condition, the time for digital devices to detect AF after stroke seems very close.

## Introduction

One quarter of all ischemic strokes (IS) and transient ischemic attacks (TIAs) are of cardioembolic origin, with atrial fibrillation (AF) being the main cause. In 20 to 30% of cases, AF is known before the stroke (1). For the remaining patients, the search for asymptomatic paroxysmal atrial fibrillation should be performed as soon as the patient arrives at the stroke center. ECG at the time of admission and more prolonged ECG monitoring can detect new AF in approximately one quarter of patients with IS (2). Identification of AF allows optimization of secondary prevention treatment by instituting oral anticoagulant therapy, which can reduce the risk of stroke recurrence by up to two thirds (3). In current practice, a main issue is that the screening strategy is based not only on scientific recommandations but also on local resources.

Detection of atrial fibrillation begins on admission of a stroke patient with a 12-lead ECG, followed by repeated ECGs, scope monitoring or telemetry during hospital stay and a Holter ECG of at least 24 h (4). Long-term cardiac rhythm monitoring is recommended in patients with cryptogenic stroke (CS) and negative initial workup (4). The longer the duration of monitoring, the higher the percentage of AF diagnosis, around 30% at 3 years for patients with implantable loop recorder (ILR) (5). Many barriers complicate the current pathway of detecting AF in stroke patients. Despite some issues, the advantages of digital devices make them a serious alternative to improve AF detection in this high-risk population.

## Current pathway for stroke patients to detect AF

**Figure 1:**
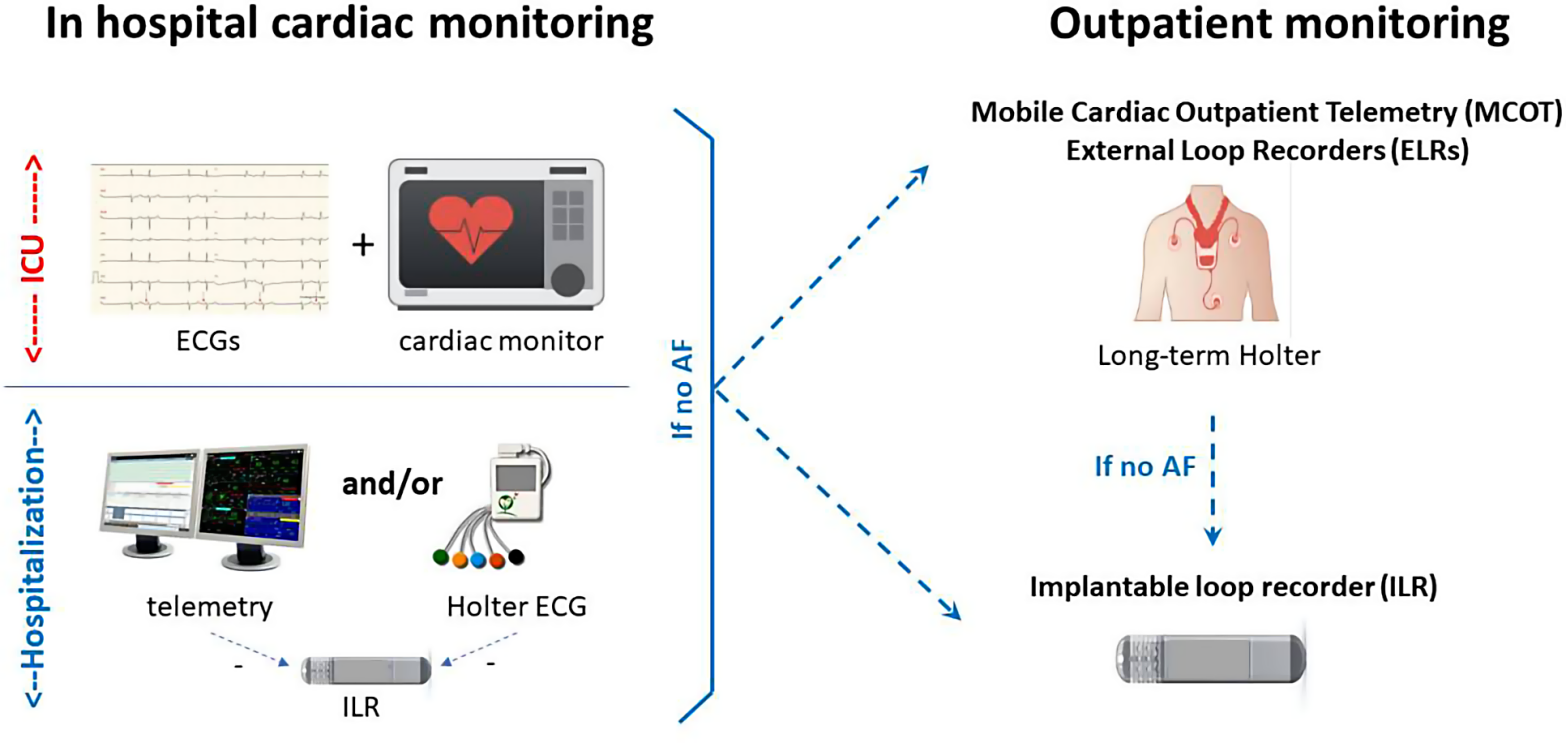
Current pathway for stroke patients to detect AF. ICU, intensive care unit.

When a patient is admitted to a stroke unit, tests are performed to determine the cause(s) of the stroke. The patient has an ECG on arrival and is continuously monitored by a cardiac monitor during their stay in the intensive care unit (ICU). After the ICU, the patient is transferred to a conventional neurological inpatient unit and monitoring continues. At this stage, the screening strategy is agreed between neurologists and cardiologists to determine the appropriate tests for the patient. However, the fluidity of this assessment depends on local organization, and the issues of this screening are threefold: the availability of monitoring equipment, the selection of patients to be proposed for long-term monitoring, and the level of benefit expected for the patients. Usually, inpatient monitoring during conventional hospitalization can be telemetry or, in case telemetry is not available, ECG Holter (more or less prolonged). However, if there is a strong suspicion of AF and depending on the local organization, ILR may be discussed before discharge. Outside of this case, after hospitalization and depending on the data from the first monitoring, ambulatory long-term monitoring is discussed using ILRs as well as mobile cardiac outpatient telemetry (MCOT), external loop recorders (ELRs) placed after ILRs in recent guidelines (6) (Figure 1).

## Current practical issues for AF detection in post stroke patients

### Main barriers

Many practical barriers exist in screening for AF in patients post-stroke. Economic issues may limit telemetry monitoring in neurology departments. The availability of Holter devices and event recorders is not always high. Appointment times for 24-h Holter ECGs and even more for external loop recorders are often long. This results in many patients not receiving the necessary tests for AF detection. This is not a recent problem: the Ontario Stroke Registry for patients managed between 2003 and 2013 found a 30% rate of 24-h Holter ECGs performed and less than 1% for longer Holter duration (7) whereas this was not the case for cardiac ultrasound. AF detection is far from optimal today too, with a use of ECG monitoring in around 10% of post IS patients in a Danish nationwide cohort, not correlated with risk factors of AF raising the appropriateness of screening (8). The use of ILR in daily practice is limited to a subset of patients, estimated at 15% in a US cohort of nearly 13,000 patients with CS (9).

### French experience

A recent national survey of vascular neurologists and heads of stroke units in France (10, 11) was conducted using structured online questionnaires. The objective was to evaluate the methodology of AF screening and to analyze (qualitatively and quantitatively) the availability and current use of AF screening in stroke units. Regarding the availability of cardiac rhythm screening, continuous cardiac monitoring during hospitalization of a stroke patient is considered necessary by 90% of neurologists, but only 1/3 of them have continuous cardiac recording monitoring (outside the intensive care unit.). In-hospital AF screening also relies, to a variable extent among centers, on initial and then repeated ECG (29%), and 24-h Holter ECG (70%). All vascular neurologists in this study considered ambulatory cardiac monitoring to be of great interest or necessity. When the 24-h Holter recording is initially normal and AF is strongly suspected, additional prolonged monitoring is suggested. 75% of neurologists request noninvasive ambulatory monitoring for at least 7 days, and more than half request ILR. The accessibility of ambulatory monitoring modalities is ranked as follows: fairly easy for 24/48h-Holter ECG (85%) and ILR (68%); fairly difficult/impossible for 3–7 days Holter ECG (51%), 8–21 days Holter ECG (75%), or e-ECG tools (99%). It is noteworthy that the ambulatory 24-h Holter ECG is obtained within one week to one month after the stroke in 70% of cases. The main barriers to developing monitoring capabilities in the SUs are lack of manpower (80%), effective network with cardiologists (56%), familiarity with techniques (42%); and cost of technical equipment (44%). This survey shows the lack of a uniform strategy regarding the methods used and their access for AF screening. These results call for the harmonization of practices and the promotion of a plan to improve AF detection (patient selection, tools, and prioritization of examinations) after an IS in France.

### Selection of patients for the screening strategy

Age, patient's cardiovascular risk factors, atheromatous disease are predictive factors for AF after stroke. The CHAD2DS2-VASC score includes these parameters (4, 12). Echocardiographic features and biomarkers—left atrial dilatation, BNP and pro-BNP (2)—and stroke due to proximal occlusion of an intracerebral artery (and therefore associated with significant neurological deficit on the NIHSS score) (13) are also predictive of AF after stroke. AF risk prediction scores have been evaluated to determine which patients with cryptogenic stroke should be offered priority for long-term monitoring. These composite scores are based on clinical, ECG, echocardiographic, and/or biological parameters to predict AF after IS but their lack of sensitivity and specificity make them difficult to use in clinical practice (14–17).

On the other hand, although the benefit of anticoagulation in secondary prevention is widely recognized (4), it is not certain that this benefit is present for patients with a very limited AF burden and it is therefore not certain that there is a need to detect very short and very rare episodes of AF (18–20). It is sometimes difficult to establish a link between stroke and AF episodes detected very long after its occurrence (21).

## Potential benefits and issues using digital devices in stroke patients

Digital devices to monitor heart rhythm can be divided in two ways (Table 1). First according to the technology used to evaluate heart rhythm, devices are electrocardiogram (ECG)-based or non ECG based including photoplethysmography (PPG). Using a non ECG based device needs confirmation via ECG and clinician oversight to confirm AF diagnosis. Second according to the mode of heart rhythm recording, the devices are wearables such as smartwatch using PPG, patches, biotextiles, belts or non-wearables such as handheld ECG, smartwatch ECG, contactless video PPG (22). The use of digital devices in the context of stroke patients therefore seems interesting because of the availability and low cost of the equipment with remote monitoring capability as well as their ease of use in hospitals, rehabilitation centers or at home and their acceptability by patients and healthcare professionals (HCP) (23). Age is not a barrier to the use of these devices in large studies (20, 24, 25). In a recent survey, more than 85% of HCP agreed that reimbursement should be applied for the clinical use of digital devices, also in the post-stroke setting (26). However, it is important to emphasize that digital devices are not yet included in the recommendations on AF detection after stroke. The lack of evaluation and of a general framework of requirements as for ambulatory ECG systems (27) make general recommendations difficult (28, 29). It is indeed essential to know for each device its sensitivity and specificity in terms of detection and diagnostic algorithms (29, 30). For example, validation studies using Holter ECG as controls reveal that chest belt devices have superior performance (accuracy of >0.90) compared to PPG-based wrist-worn devices (highly variable accuracy range, 0.36–0.99) (22). However, given the limitations in terms of access to care, budgetary constraints, and the incomplete level of evidence for cardiac rhythm monitoring after stroke, it seems essential to evaluate the benefit of using these digital devices to address these concerns. Conventional monitoring combines admission 12-lead ECG, repeated ECGs, scope monitoring and/or telemetry in the neurovascular unit, then Holter ECG from 24 h to 7 days, and finally, depending on the estimated probability of AF, long-term monitoring, preferably with ILR (1, 6). At each stage of monitoring, digital devices could play an alternative or even substitute role (Table 2).

**Table 1:** Digital devices to monitor heart rhythm, according to their technology and the mode of heart rate recording. Using a non ECG-based device needs confirmation via ECG.

	Wearable	Non wearable
ECG-based	Patch, vest (biotextiles), belt	Handheld ECG, smartwatch-ECG
Non ECG-based (including PPG)	Smartwatch-PPG	Contactless video PPG

PPG, photoplethysmography.

**Table 2:** Current pathway of stroke patient with equipment use for AF detection, and potential alternative by digital devices.

Stroke patient's pathway	Current monitoring	Digital devices as possible alternative
Admission in stroke unit	12-lead ECGs	1. ECGs acquired through wearable devices (ex: patch-type wireless 12-lead ECG) 2. Systematically digitized ECG [AF prediction in sinus rhythm (AI) and/or ECG marker of atrial cardiopathy]
Stroke unit and neurology department	Serial ECGs Scope monitoring Telemetry In patient 24-h Holter	1. PPG-based monitoring device—Wearable wireless devices (watch) HD video camera in room, cameras from smartphone/tablets 2. ECG-based monitoring devices (handheld devices or wearable wireless devices such as biotextiles, belt, watch)
Outpatient shot term	24-h/7 days Holter	1. Adhesive single-use patch: up to 14 days of continuous recording with a single or two leads ECG 2. ECG recordings through connected devices (handheld, watch)
Outpatient long term	MCOT External loop recorder	1. Sequential ECG recordings through wearable devices (watch) or continuous ECG recording (biotextile) 2. Continuous PPG-based with wearable devices or smartphone/tablet cameras
Outpatient very long term	Implanted loop recorder	Initial phase to better select patient for ICM implantation or alternative? 1. Sequential ECG recordings through wearable devices 2. Continuous PPG-based wearable devices or smartphone/tablet cameras

## Digital devices as alternative to standard equipment at each stage of stroke patient pathway

**Figure 2:**
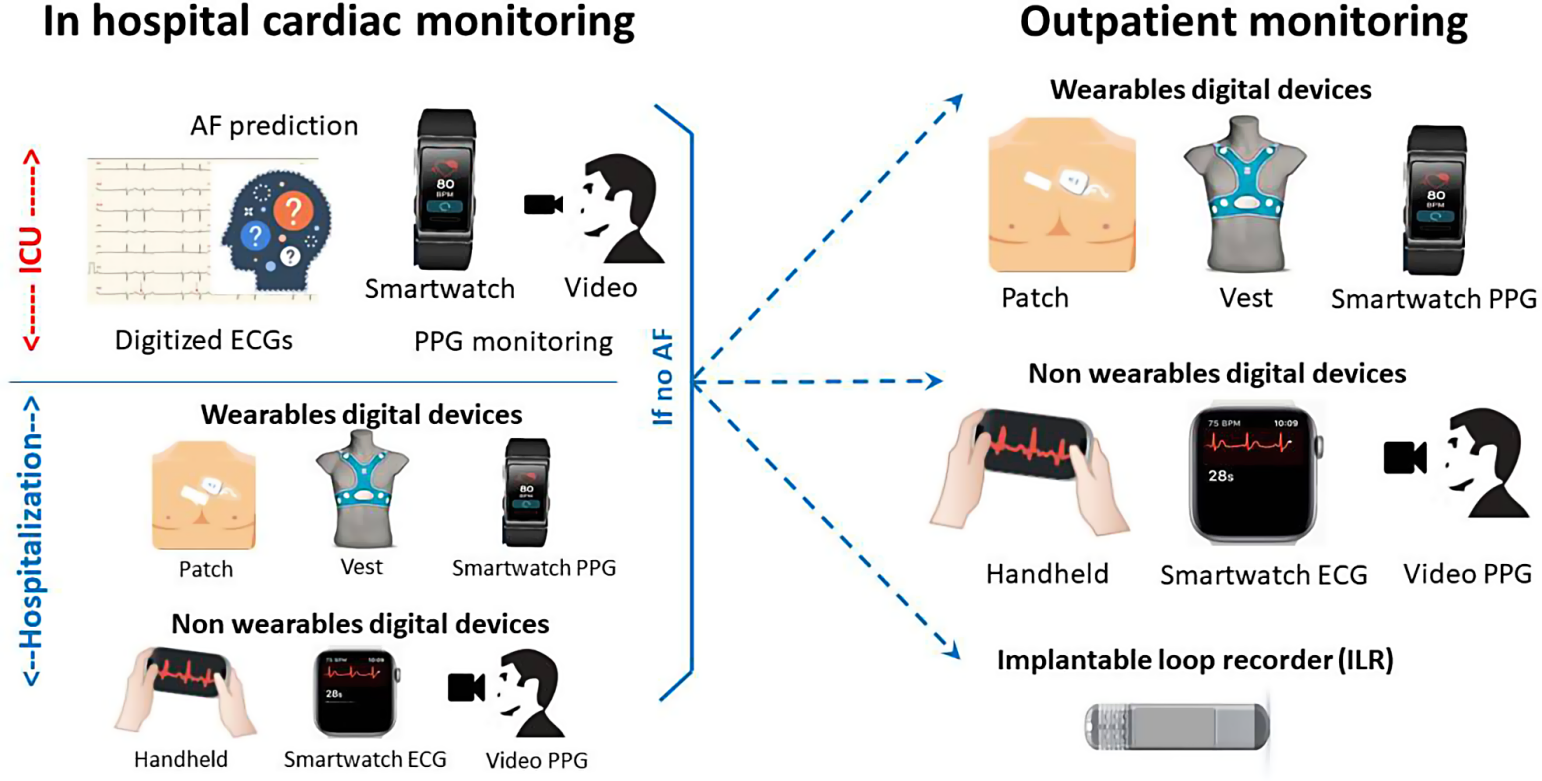
Digital devices as an alternative for stroke patients to detect AF.

The 12-lead ECG on admission is mandatory to detect AF and sometimes conduction disorders or to suspect underlying heart disease. AF detection rate is around 7.7% in stroke patients without known AF (1). Simplification of ECG acquisition and digital processing could provide potential benefits in clinical practice. New systems are currently developed such as a patch-type wireless 12-lead ECG (31) allowing a layperson to acquire a 12-lead ECG in a median time of 3 min. Currently, digital processing of ECGs seems mandatory to store them, transfer them for analysis by a cardiologist directly or after triage through a dedicated algorithm (32). Using artificial intelligence algorithms (33) or particular ECG measurement (34), recent publications suggest a potential value of ECG analysis in sinus rhythm to predict AF occurrence and/or stroke risk. A higher level of evidence is needed but these potential uses reinforce the need for routine ECG digitization in daily practice (Figure 2).

Classically, four different types of monitoring are used on stroke units and neurology wards to detect AF providing a 5.1% rate of AF detection: serial electrocardiography, continuous inpatient ECG monitoring, continuous inpatient cardiac telemetry and in-hospital Holter monitoring (1). Serial electrocardiography could be performed in an easier mode than a standard ECG machine using a single-lead connected device with a high sensibility and specificity (35). In the SPOT-AF study, patients were monitored using a smartphone-enabled handheld ECG (iECG) during routine nursing observations, and underwent 24-h Holter monitoring according to local practice. AF was detected in 25/294 (8.5%) by iECG, and 8/294 (2.8%) by 24-h Holter recordings (non-randomized comparison) (36). Other techniques could be evaluated by comparison to scope monitoring and telemetry such as continuous photoplethysmography (PPG)-based wearable devices providing a cheap and leadless solution easier to handle in daily practice. Using facial video cameras from smartphone or tablets for measurement of pulse rate and AF detection is currently under evaluation (37). However it is important to remember that detection of AF based on PPG currently requires confirmation of AF by ECG (29). Finally, continuous ECG monitoring is taking part of bedside AI-based predictive analytics monitoring (38) that could be useful for post stroke patient management in the future.

Ambulatory Holter monitoring from 24 h to 7 days provides a 10.7% rate of AF detection (1). Digital devices such as single ECG patch monitor providing up to 14 days of recording have been developed to replace conventional Holter ECG with leads. A randomized controlled trial of 116 patients following stroke showed superiority compared to a 24-h Holter monitor (detection of 1 participant in the Holter monitor group compared to 8 participants in the patch group) (39). This patch is currently recommended by the National Institute for Health and Care Excellence in the UK as an option for people with suspected cardiac arrhythmias who would benefit from ambulatory ECG monitoring for 24 h. Another approach currently under investigation is the continuous monitoring of PPG-based rhythm for weeks after stroke: in the Liverpool Huawei stroke study effectiveness, cost-effectiveness and patient and staff acceptability of using Huawei smart wearables to detect AF following IS during four weeks post discharge will be determined in 1,000 stroke patients (40). Signals will be analyzed through remote monitoring and patients with suspected AF will be referred to a cardiologist. In the multicenter CryptoAF study (41), another wearable device, a textile wearable holter monitoring, have been tested up to 90 days, detecting a high percentage of AF, although a significant number of patients did not complete the monitoring. A self-screening procedure using a patch-ECG could be also an interesting approach as recently demonstrated in individuals aged more than 65 years from the general population of Norway (42).

Ambulatory long-term monitoring using MCOT, ELRs and ILRs provides a 16.9% rate of AF detection (1). External monitoring is sometimes proposed before ILR. The randomized CANDLE-AF study will evaluate a 72-h single-patch monitor to standard strategy and to an event-recorder-type device in 600 IS patients without any history of AF (43). Single-patch monitor arm will repeat monitoring at 1, 3, 6, and 12 months, event-recorder-type arm will repeat monitoring twice daily for 12 months. Recent studies have shown the superiority of ILR on ELR in post-stroke AF detection (44). ILR is preferred upon MCOT and ELRs in recent guidelines (6). A predischarge nurse-led implantation of ICM has been the subject of specific patient pathway leading to short delay (45) but the follow-up and analysis of electrograms remain a significant workload despite the development of remote monitoring and the use of artificial intelligence algorithms (46). Moreover, the cost of ILR is quite high, although below the limit of acceptability for cost-effectiveness (47, 48). The constant loop recording of ECG of ILR for around three years explain its high yield of AF diagnosis compared to other techniques. Recently, in a sub-study of LOOP study in 590 patients aged more than 70 years followed for 3 years, different types of sequential screenings from 10-second ECG recording every day for 14 days to annual 30-day monitoring were applied. Even with the more intense screenings, more than 4 in 10 patients with AF and around one in six with underlying ≥24-h episodes will go undetected (49). Except particular case (50), it seems unlikely that any connected tools used in a sequential way could provide a high AF diagnostic yield such as ILRs. However, combination of continuous PPG-based monitoring with wearables devices such as belts, watch or ring-types and sequential ECG-based monitoring with the same wearable devices could be an interesting alternative to compare to ILR. This combination is currently being investigated in the Heartline randomised trial in people over 65 years-old using a smartwatch connected to a smartphone compared to using a smartphone app only (51).

The detection of AF in patients with CS is an essential part of management to limit the risk of recurrence. In practice, not all patients who need AF screening do so, or with significant delays. The disparities of access to examinations, their costs as well as the increasing workload require an evolution of practices both in terms of organization and the type of equipment used. The ubiquity and ease of use of digital devices, together with their evaluation in large population and their expected lower cost, make them attractive as potential alternatives to current equipment at all stages of patient management. However, reliability and accuracy of each device for the detection of paroxysmal AF in patients with CS must be established before inclusion in clinical practice is considered as well as the actual impact on workload. Under this condition, the time for digital devices to detect AF after stroke seems very close (52).
